# PEEK for Oral Applications: Recent Advances in Mechanical and Adhesive Properties

**DOI:** 10.3390/polym15020386

**Published:** 2023-01-11

**Authors:** Chengfeng Luo, Ying Liu, Bo Peng, Menghao Chen, Zhaogang Liu, Zhanglong Li, Hai Kuang, Baijuan Gong, Zhimin Li, Hongchen Sun

**Affiliations:** 1 School of Stomatology, China Medical University, Shenyang 110002, China; 2 Liaoning Provincial Key Laboratory of Oral Diseases, Shenyang 110002, China; 3 The Affiliated Taian City Central Hospital of Qingdao University, Taian 271000, China; 4 Department of Oral and Maxillofacial Surgery, College of Stomatology, Guangxi Medical University, Nanning 530021, China; 5 Key Laboratory of Research and Application of Stomatological Equipment (College of Stomatology, Guangxi Medical University), Education Department of Guangxi Zhuang Autonomous Region, Nanning 530021, China; 6 Hospital of Stomatology, Jilin University, Changchun 130021, China

**Keywords:** adhesive, dental application, dental prostheses, mechanical properties, polyetheretherketone, surface treatment, 3D printing

## Abstract

Polyetheretherketone (PEEK) is a thermoplastic material widely used in engineering applications due to its good biomechanical properties and high temperature stability. Compared to traditional metal and ceramic dental materials, PEEK dental implants exhibit less stress shielding, thus better matching the mechanical properties of bone. As a promising medical material, PEEK can be used as implant abutments, removable and fixed prostheses, and maxillofacial prostheses. It can be blended with materials such as fibers and ceramics to improve its mechanical strength for better clinical dental applications. Compared to conventional pressed and CAD/CAM milling fabrication, 3D-printed PEEK exhibits excellent flexural and tensile strength and parameters such as printing temperature and speed can affect its mechanical properties. However, the bioinert nature of PEEK can make adhesive bonding difficult. The bond strength can be improved by roughening or introducing functional groups on the PEEK surface by sandblasting, acid etching, plasma treatment, laser treatment, and adhesive systems. This paper provides a comprehensive overview of the research progress on the mechanical properties of PEEK for dental applications in the context of specific applications, composites, and their preparation processes. In addition, the research on the adhesive properties of PEEK over the past few years is highlighted. Thus, this review aims to build a conceptual and practical toolkit for the study of the mechanical and adhesive properties of PEEK materials. More importantly, it provides a rationale and a general new basis for the application of PEEK in the dental field.

## 1. Introduction

Trauma, fractures, periodontal disease, and caries have led to an increasing clinical demand for high-performance restorative materials in modern dentistry [1,2]. Materials traditionally used for dental and maxillofacial restorations mainly include ceramics and metallic biomaterials. Ceramic materials are widely used because of their good aesthetic properties, robustness, and comfort [3]. Metallic biomaterials such as cobalt-chromium (CoCr) alloys, titanium (Ti), and some titanium alloys are often used as permanent or temporary implants due to their high mechanical strength and corrosion resistance [4]. However, ceramic materials mostly contain feldspar, resulting in a lower strength and higher brittleness. The high elastic modulus of metal implants often leads to the “stress shielding” effect, which can result in osteoporosis or even implant failure in long-term applications. Moreover, metal ions may cause gum discoloration and allergic reactions in some patients [5]. All these disadvantages are a motivation for improving materials and developing new materials.

Polyaryletherketone (PAEK) is a crystalline polymer formed by linking phenylene rings through oxygen bridges and carbonyl groups (ketones). According to different structures, PAEK mainly includes polyetherketone (PEK), polyetheretherketoneketone (PEEKK), polyetherketoneetherketoneketone (PEKEKK), polyetherketoneketone (PEKK), and polyetheretherketone (PEEK) [6]. As a high-performance plastic polymer with semicrystalline properties, PEEK has become a hot topic for new material research due to its good biocompatibility, high-temperature resistance (melting point of 343 °C), excellent fatigue properties, high toughness, relatively low wear rate (0.9 ± 1.1 mm^3^/MC), corrosion and aging resistance, ease of processing, and color stability [7]. Additionally, compared to zirconia and metal alloys, PEEK does not cause metal allergies and has X-ray radiolucency [8]. Thus, it allows patients to undergo routine examinations such as CT scans and MRIs without image artifacts [9,10].

PEEK exhibits excellent mechanical properties and a greater lightness compared to conventional materials. Its modulus of elasticity (3–4 GPa) is similar to human bone tissue (14 GPa) [11], which provides a damping effect for PEEK restorations [7] and reduces stress shielding. However, fewer reviews have comprehensively summarized the research advances in PEEK for a variety of dental applications. The stiffness of PEEK may not be sufficient to withstand load-bearing stresses [12] and there is a higher risk of fracture. However, PEEK can be blended with materials such as fibers and ceramics to improve its mechanical strength and provide advantages in various dental applications. Recently, 3D printing has been used to manufacture PEEK scaffolds and prostheses. This technique can produce more precise and complex end products [13]. However, in contrast to materials such as metals, evidence is needed to determine the mechanical properties, accuracy, and precision of prostheses made of PEEK [14] and to help select the best processing techniques and parameters. In addition, compared to ceramic materials such as zirconia, the PEEK prosthesis fails to achieve satisfactory aesthetic outcomes unless veneered with composite resins [15,16]. Yet, its inert surface makes it difficult to bond PEEK with composite resins and abutment teeth, limiting its clinical application. PEEK must undergo surface treatments such as roughening [17], the introduction of chemical groups [18], and adhesive systems to improve its adhesive properties. The most effective PEEK bonding strategy has yet to be established.

Therefore, this review summarizes the state-of-the-art mechanical and adhesive properties of PEEK, particularly in regard to dental applications. It describes the progress of research on the mechanical properties of PEEK in the fields of dental implant treatment, fixed dental prostheses, removable dental prostheses, maxillofacial prostheses, and orthodontic treatments. It also outlines research advances in the mechanical properties of PEEK composites reinforced with different materials such as fibers and ceramics. The effects of different techniques, such as pressing, milling, and 3D printing, on PEEK properties are also summarized. Additionally, this paper focuses on the effects of surface treatments and adhesive systems on the adhesive properties of PEEK.

## 2. Performance Requirements for Medical Materials

The ideal material for medical repair and bone grafting requires adequate mechanical strength and good tensile, flexural, and compressive moduli. Mechanical strength is a fundamental property of any material that is used as a long-term implant in the oral cavity. Additionally, a good modulus of elasticity prevents the “stress shielding” of the bone, i.e., bone destruction and resorption at the healing site [7]. In addition to mechanical properties, good biological properties are also critical for biomedical applications, such as biocompatibility and osteogenic properties. In terms of physical and chemical properties, medical materials should have certain surface properties and corrosion and aging resistance. Surface properties mainly include roughness, surface tension, and Martens force/depth indentation, which includes, namely, hardness (HM), modulus (E_IT_), and creep (C_IT_). Among them, hardness is an important parameter for material durability [19], whereas roughness and surface tension are key factors of material bonding properties. The magnitude of bond strength can affect the retention, edge fit, microleakage, and longevity of indirect restorations. Table 1 summarizes the performance requirements and relevant test methods for medical materials.

## 3. Mechanical Properties of PEEK in Dental Applications

There are numerous types of medical materials currently used in dentistry, with different performance advantages and disadvantage**s** (Table 2). PEEK has been increasingly studied in dental applications, such as in oral implant treatments (oral implants and implant abutments) [31], restorative dentistry(crowns, fixed and removable dentures, and posts and cores), maxillofacial prosthetics, and other oral applications (orthodontic wires and retainers and scaffolds for cartilage repair) [14,32]. For better use in dentistry, the mechanical properties of PEEK (Table 3) can be improved by adding materials, such as fibers, carbon nanomaterials, and ceramics, or by improving processing techniques and parameters.

### 3.1. PEEK as an Oral Implant Material

#### 3.1.1. Implants Made of PEEK

Implant dentistry has become an increasingly common and effective treatment for partial or complete edentulousness. Titanium is the most commonly used material for dental implants. Because PEEK has similar osteoconductive properties to titanium and an elastic modulus close to that of human bone [52], PEEK has been suggested to replace titanium as a dental implant material. There are many factors affecting implant stability, such as occlusal load, bone quality and quantity, peri-implantitis, and marginal bone loss (MBL) [53]. The study by Ouldyerou et al. [54] showed that conventional titanium implants transfer a small amount of micromovement, with stress concentrations occurring only in the apical region of the cancellous bone. However, despite bone loss, a Ti-PEEK composite implant showed a larger stress distribution in the surrounding bone region and was superior to conventional titanium implants in reducing bone resorption (stress shielding). In addition, animal studies have found that Ti-coated PEEK exhibits more osteogenic behavior than uncoated PEEK [55]. Uncoated PEEK is highly hydrophobic and may prevent initial cell adhesion. One study [56], nevertheless, found increased bone–implant mechanical interlocking with porous PEEK compared to smooth PEEK and Ti-coated PEEK, showing improved cellular osteogenic differentiation and increased implant osseointegration. In addition to Ti-PEEK composites, PEEK was also modified with hydroxyapatite (HA) for use as implants. However, the addition of bioactive HA particles in the size range of 2–4 mm can negatively affect the mechanical properties (tensile strength) of PEEK [51]. Implants prepared from PEEK nanocomposites have the advantages of increased bioactivity and improved mechanical properties [57,58]. The presence of surface roughness on the micron and nanoscale promotes cell adhesion. The osteoconductivity and bioactivity of unmodified PEEK are insufficient for use as dental implants. More research and long-term trials are needed before PEEK can be used as a dental implant, with a focus on improving the bioactivity of PEEK.

#### 3.1.2. PEEK Implant Abutments

**Figure 1:**
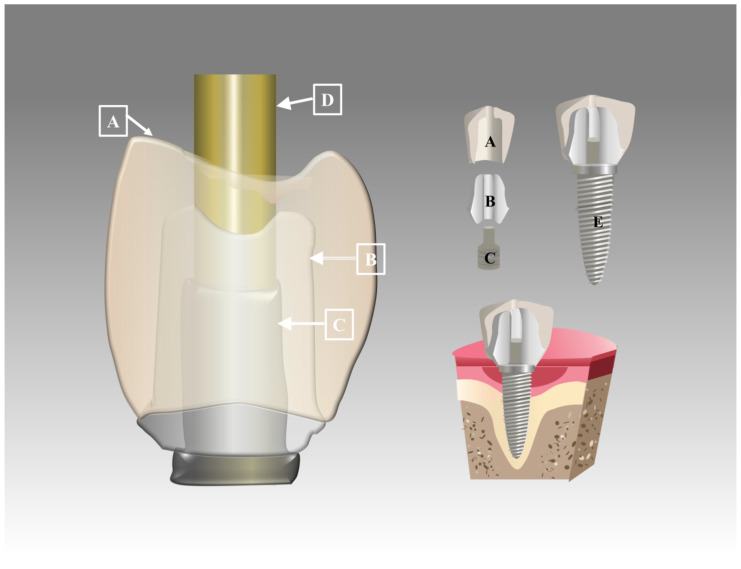
Schematic diagram showing design of abutment and crown: A—crown; B—abutment; C—insert; D—screw channel; E—implant.

An abutment is the connecting element between the prosthetic restoration and the implant in implant dentistry (Figure 1). Currently, known abutment materials mainly include pure Ti or Ti alloys, CoCr alloys, alumina (Al_2_O_3_), zirconia (ZrO_2_), the polymer-infiltrated ceramic network (PICN), polymethyl methacrylate (PMMA), and PEEK [34]. Among them, PEEK has been advocated for as an alternative to metal components, especially for the manufacture of temporary abutments [7]. A study [31] showed that titanium grade 5 is superior to PEEK abutments with regard to resistance to torque loss and microleakage. This may be a result of the high incidence of the plastic deformation of PEEK abutments [59]. However, PEEK abutments may be suitable for temporary restorations in patients without parafunction, especially in anterior tooth defects [31]. Apart from this, a custom PEEK healing abutment required fewer restorative steps to create the desired gingival emergency contour during the surgical phase than using a standard healing cap [7,60]. The PEEK custom healing abutment can be adjusted to fit the implant site by adding or reducing the contour intraorally [61]. However, studies comparing the effectiveness of custom healing abutments with different materials are still lacking [62] and further studies should be conducted in the future.

The occlusal load and stress distribution patterns during the implant healing phase are key factors affecting the long-term outcomes of implant treatments. Taha et al. [63] investigated the effect of different combinations of crowns and custom abutments on the force absorption capacity in implant-supported restorations and concluded that the combination of resin-based ceramic crowns and PEEK abutments did not enhance the force absorption. Possibly due to the significantly lower elastic modulus of PEEK custom-made abutments as compared to zirconia abutments, lower stress values were generated within the abutment structure itself, but the lack of supporting force resulted in more stress on the overlying crown [64]. Mourya et al. [65] analyzed the stress distribution around the abutment at different angles under vertical and oblique loading for titanium and carbon-fiber-reinforced PEEK (CF/PEEK) implants. Studies suggest that molar patients can receive straight abutments combined with PEEK crowns to reduce intraosseous stress concentration and prevent implant failure. When considering the long-term results of implant treatments, the effect of biological aging on implant surfaces cannot be ignored. It was suggested that UVC photofunctionalization can be an effective method to remove carbon contamination compounds from the surfaces of titanium dioxide (TiO_2_), ZrO_2_, and PEEK abutment materials to reverse the adverse effects of biological aging in dental implantology [66].

The available evidence suggests that PEEK abutments do not have sufficient biomechanical requirements to replace established titanium abutments [67], and zirconium remains the most biocompatible abutment material [68]. However, as mentioned before, PEEK materials offer specific advantages in applications such as temporary restorations in patients without functional impairments and the creation of emergency contours during the surgical phase, especially for implant restorations in patients carrying weak stress loads.

### 3.2. PEEK as an Oral Prosthesis Material

Studies have shown PEEK to be a potential restorative material when used in the oral cavity [69]. In mechanical property studies related to the field of dental prosthetics, PEEK materials are typically divided into three categories based on their composition: pure PEEK, CF/PEEK, and other PEEK composites.

#### 3.2.1. PEEK

Light-curing resins are commonly used as fillers and restorative materials in dentistry [70], and PMMA is a commercial photocurable resin used in the 3D printing industry, which has the advantages of low odor, low irritation, good flexibility, and low cost [71]. After aging in different solutions, PEEK demonstrated the lowest solubility and water absorption values compared to composite resins, a hybrid material, and PMMA-based materials and demonstrated similar hardness parameters as those of PMMA-based materials [72]. In addition, PEEK does not shrink during the polymerization process as composite resins and PMMA do [39], which facilitates its use in dental prosthetics.

PEEK can be used for fixed dental prostheses (FDPs), for example, being made into crowns [12], frameworks, posts and cores, etc. Schmeiser et al. [24] simulated the effect of the geometry of milled crowns (PEEK, PMMA, and silicate ceramic (SiO_2_)), crown-abutment material combinations, and thermal loading on the in vitro wear of crown materials during mastication and found that thermal loading did not affect material loss. PMMA crowns showed the highest material loss and PEEK had the lowest material loss. Moreover, PEEK exhibited the lowest marginal (56.00 ± 4.67 μm) and internal clearance values (128.90 ± 8.39 μm) and the greatest fracture resistance (840.90 ± 13.23 N) compared to polylactic acid and PMMA in vitro [73], and, thus, it is recommended as a material for provisional crown restorations [12]. As a framework, the PEEK material can be veneered with a composite resin as an implant-supported FDP for patients with metal allergies [45]. The average fracture strength of crowns obtained by laying composite materials on a PEEK coping (2134.64 MPa) was significantly higher than that of zirconia composite crowns (1142.3 MPa) [74]. Moreover, Tasopoulos et al. [75] performed a successful bilateral restoration of mandibular first molar teeth using a modified PEEK inlay-retained resin-bonded fixed dental prosthesis (IRRBFDP), and no debonding and fracture of the framework or loss of retention was observed. The low modulus of elasticity of PEEK can lead to lower root fracture rates and help protect the tooth structure. Therefore, PEEK can also be fabricated into posts and cores, becoming a viable alternative to rigid casts or zirconia posts, or even fiber-reinforced composite posts, especially when combined with lithium disilicate crowns (LDC) [76]. When restored with glass fiber (GFP) or PEEK posts and LDC, the roots of maxillary central incisors exhibit a similar stress distribution under occlusal loading [77]. Yet, the use of prefabricated fiberglass posts for teeth with flared root canals is controversial. Fiberglass posts are a mixture of glass and resin with multiple interfaces, and moisture in the oral cavity may result in the reduced strength of the post. However, tubular fiberglass sleeves are thought to strengthen the area around the post space [78]. For flared root canals, a combination of PEEK posts and fiberglass sleeves is recommended based on the results of loading experiments [79].

Applications of PEEK as removable dental prostheses include removable partial dentures (RPDs) (frames and clasps), telescopic crown dentures [80], and obturators [14]. Compared to PMMA, partial denture frames made of PEEK with notches for labial and buccal ties or special designs may be less prone to breaking because of their higher Izod impact strength. Meanwhile, PEEK provides a higher Young’s modulus but lower flexural deformation than PMMA, which may reduce the load applied to the underlying tissue, thereby minimizing the possibility of relining the substrate after a few weeks [81]. To achieve the fixation of RPDs, the tip of the clasp fixation arm must have a sufficient undercut below the contour height [82]. A previous study found no significant difference in the flexural load and the deflection of the retentive force at 0.50 mm between PEEK and CoCr materials [83]. However, Zheng et al. [84] showed that the flexural load of PEEK specimens was lower than that of cast and laser-sintered CoCr specimens. Similarly, the results of constant displacement fatigue tests on three shape-optimized PEEK clasps and one standard shape Co-Cr alloy clasp showed that the average load values of PEEK were significantly lower than that of the Co-Cr alloy [85], but the study concluded that the retention force provided by the shape-optimized PEEK clasp was still adequate for clinical use (5–10 N) [86] and there was no significant difference in the long-term deformation between the two materials. In contrast to the results of this study, Tribst et al. [87] believed that polyoxymethylene and PEEK are not suitable for use as clasps because the maximum stress (189.9 MPa) that occurs when they are removed at a high level of undercuts (0.75) is higher than the material strength (80 MPa). The reason for the different results may be that the shape of the clasps was not optimized in the later study. Analysis of shape-optimized clasps by finite element methods showed that the maximum stress concentration always exists at the bottom of the specimen, and the influence of clasp thickness [88] and taper on the average load value is greater than that of clasp width [85]. Compared with standard alloy clasps [83], the shape-optimized PEEK clasps exerted less pressure on the abutment teeth, provided adequate retention, and met aesthetic requirements, suggesting that PEEK is a promising alternative to conventional metal retainers. Aside from RPDs, PEEK can be used as telescopic crown dentures with a comparable performance to commonly used material pairs [80]. It can provide additional security against the possible loss of retention during restoration or refilling, which may require further research.

#### 3.2.2. CF/PEEK

PEEK suffers from several disadvantages that limit its application, such as an insufficient mechanical strength, lack of secondary processing capabilities, and poor bioactivity. However, this situation can be improved by adding carbon fibers (CF), carbon nanofibers (CNF), bioactive glass (BG), HA [44], and other materials to the PEEK matrix to create composites [89]. CF/PEEK is formed by adding carbon fibers of varying lengths and weight fractions (mainly consisting of 60 or 30 wt.%) to PEEK and offers a higher mechanical strength and wear resistance than conventional PEEK materials [90]. The elastic modulus of the CF/PEEK material can vary with the length and thickness of carbon fibers and is controlled in the range of 3.5–58.5 GPa, which is closer to the elastic modulus of human bone (13.7 GPa); thus, it provides a good mechanical adaptability. In particular, the CF/PEEK material has a density of 1.3 g/cm^3^, which reduces the weight of the prosthesis, facilitating immediate postoperative stability. In addition to its mechanical advantages, the light transmission of CF/PEEK is unmatched by most metallic materials [91]. However, PEEK has a high melting temperature and a large melt viscosity, which makes it more difficult to bond it to the fibers. Therefore, interlaminar interface cracking or delamination is one of the most common types of failure in laminated fiber-reinforced composites during the clinical service life [40].

Fused deposition modeling (FDM) (i.e., fused filament fabrication (FFF)) 3D printing technologies offer unique advantages for the rapid prototyping of thermoplastics [92]. Due to the high thermal conditions (380–440 °C) used in the FDM process for fiber-reinforced PEEK composites [93], a fiber content above 20 wt.% in CF/PEEK composites can lead to a high melt viscosity, which can result in 3D printing failure [92]. Short and continuous fiber-reinforced polymer composites are widely used in FDM to improve the mechanical and thermal properties of polymer-based materials [94]. A study [92] was conducted on 5 wt.% CF/PEEK, which found that the tensile and flexural strengths of CF/PEEK increased with higher nozzle and platform temperatures, likely because of the better melt flow and formability of the printed material at higher nozzle temperatures. In addition, higher platform temperatures generated more energy, which improved penetration and diffusion between the filaments and interlayer. However, with more fibers introduced, the impact properties of fiber-reinforced PEEK composites decreased [92], probably owing to the introduction of pores and the degradation of molecular chain properties during filament preparation [95].

**Figure 2:**
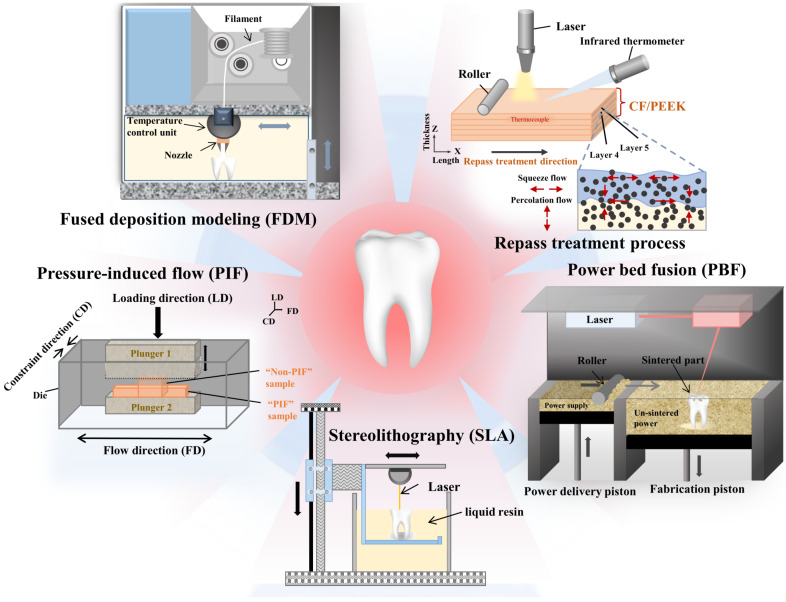
Processing techniques used for PEEK. CF/PEEK—carbon-fiber-reinforced PEEK.

Wu et al. [30] fabricated CF/PEEK laminates via laser-assisted forming with a repass treatment (Figure 2). Similar to FDM printing technology, laser-assisted forming is also a layer-by-layer laying process. Due to the repass treatment of the top surface of the laminate by laser heating and roller compaction, the squeeze flow and percolation flow of the resin, as well as the reheating of the laminate body, were generated. The repass-treated laminate has fewer voids and higher crystallinity, and its interlaminar shear strength (ILSS) can reach 66.37 MPa, which is 32.87% higher than that of the untreated laminate [30]. A post-heat treatment process can also improve the mechanical properties of FDM 3D-printed fiber-reinforced PEEK composites by improving the crystallinity and interfacial bonding properties [23]. A heat treatment at a temperature of 250 °C for 6 h reduced inter-fiber defects. Additionally, heating the printed material at 230 °C has the potential to increase the interlaminar tensile strength by more than five times, from 6.96 MPa to 36.28 MPa [96]. However, 3D printing usually favors the use of glassy polymers because semicrystalline polymers undergo a step change in viscosity during crystallization, causing the mobility of the polymer to stop, which usually deforms the printed material due to the stresses generated during crystallization [97,98]. Methods such as using improved technology to add additives to reduce crystallization [99,100] and dilute the crystalline material with fillers have been adopted to reduce the deformation of the printed material. Unlike traditional heat treatment processes, the rapid heating (10–20 s) and cooling via microwave energy results in a significant increase (250–400%) in the modulus due to the coupling of microwave energy with the carbon filler in CF/PEEK composites [97], but does not increase the crystallinity or average crystal size of PEEK and may reduce the deformation of the printed structure.

#### 3.2.3. Other PEEK Composites

In recent years, various carbonaceous materials such as carbon nanotubes (CNTs) and graphene have been utilized as reinforcing particulate materials in PEEK composites [101]. When the surface of the CF/PEEK composite was treated with concentrated sulfuric acid, a three-dimensional porous network could be formed, and the pore size of the porous layer increased alongside the increase in CF content. Through graphene oxide (GO) solution modification, filamentous GO folds were formed on the surface of the sulfonated material. Unlike the increase in contact angles of all samples after sulfonation, GO functional wrinkles significantly reduce the contact angle of the material, upregulating the surface hydrophilicity [102].

Ceramic biocomposites may contain various reinforcing particles such as TiO_2_, Al_2_O_3_, ZrO_2_, HA, tricalcium phosphate (β-TCP), and calcium phosphate (CaP). By adding 20% of a special ceramic filler to PEEK, a bioactive, thermoplastic, high-performance polymer (Bio-HPP) was obtained [103]. Nano-titanium dioxide is a semipermanent antibacterial agent, which can generate reactive oxygen species to kill bacteria under ultraviolet light. Chen et al. [104] prepared reinforced PMMA composite resins with good antibacterial activity and enhanced mechanical properties by adding TiO_2_ (1wt.%) and PEEK (1–3wt.%), in which TiO_2_ nanofillers and PEEK microfillers functioned in a collaborative manner. For implant-supported, 4-unit cantilever FDP, the TiO_2_ filler content and veneer technique of the PEEK framework had an effect on fracture load. The highest fracture resistance (4548 ± 216 N) of the restorations was achieved when a 30% TiO_2_-filled PEEK material was used and prefabricated veneers were applied. However, aging had no effect on fracture load [45]. During aging, Babaier et al. [26] found that the TiO_2_-reinforced PEEK showed slight changes in food-simulating liquids (FSLs), such as 70% ethanol, and exhibited a greater relative stability. In addition to aging resistance, the hardness of a material is an indicator for examining the durability in oral applications. Lümkemann et al. [47] found the Martens hardness (HM) parameters, which are derived from the indentation depth under a working load, improved with the increasing percentage of TiO_2_ in the PEEK matrix. The surface characteristics, such as roughness and modulus, also differed in a rough correlation with the filler content (wt.%). By adjusting the pore size and HA content, the elastic modulus of the PEEK/HA scaffold could be widely adjusted in the range of 50.6–624.7 MPa, which is similar to the range of variation in natural cancellous bone [44]. However, unlike the increase in compressive strength and modulus, the tensile and flexural strength decreased with the increase in HA content [43,44].

It is well known that amorphous calcium phosphate (ACP) has a tendency to crystallize, whereas amorphous magnesium phosphate (AMP) formed via the incorporation of Mg^2+^ ions in ACP inhibits its crystallization [105] and is thermally stable (it retains its amorphous character). Sikder et al. [106] melt-blended novel AMP particles with PEEK-based composite fibers to develop amorphous magnesium phosphate PEEK (AMP-PEEK) composite filaments. The study showed that AMP-PEEK composites have a high zero-shear viscosity and low infinite-shear viscosity, with enhanced bioactivity and osseointegration. The composites may be good candidates for the 3D printing of restorative materials for oral and maxillofacial surgery, which are worth further studies.

### 3.3. 3D-Printed PEEK in Oral and Maxillofacial Surgery

Recent advances in the development of biomaterials have provided attractive alternatives for bone grafting. Thermoplastic polymers such as PEEK, PEKK, polyphenylsulfone (PPSU), and polyethylene (PE) have smaller atomic numbers [9,10], and, therefore, produce fewer streaks and halo artifacts in CT images than titanium while maintaining a tolerable mechanical stability and biocompatibility, making their application in oral and maxillofacial surgery highly beneficial. There are two main ways to process PEEK in dentistry. One way of manufacturing PEEK is by vacuum pressing (pressing from granules or pellets), and the other is CAD/CAM (computer-aided design and computer-aided manufacturing) milling. The third stage of the CAD/CAM system involves prosthesis construction, which can be carried out using either subtractive or additive manufacturing techniques [107,108]. Among them, additive manufacturing (AM), commonly known as 3D printing, is a manufacturing technology that creates solid objects layer by layer through extrusion, sintering, melting, photocuring, and jetting [109]. After the material is manufactured, the properties can be further improved through postprocessing measures such as coating, polishing, and heat treatments.

Muhsin et al. [81] found that CAD/CAM-milled PEEK processes at a mold temperature of 200 °C outperformed pressed PEEK in terms of mechanical properties, such as tensile and flexural strength. However, using a solid-state pressure-induced flow (PIF) process (Figure 2), a bioinspired nacre-like PEEK material can be prepared [110]. The process involves the use of a mold to apply pressure to a solid material, forcing the sample to flow in one direction within the confinement of both sides. PIF-treated bioinspired nacre-like PEEK offers a unique combination of high stiffness and excellent ductility, maintaining good biocompatibility while improving mechanical matching to the surrounding tissue, which complements unfilled PEEK and conventional CF-PEEK grades. Moreover, Li et al. [111] found that the temperature and compression time of the hot compression molding process can sensitively change the mechanical properties (e.g., elongation at break) of PEEK plates; however, pressures greater than 1.5 MPa have limited effects on the mechanical properties of PEEK plates. Furthermore, a hot compression temperature of 400 °C, a compression time of 30 min, and a pressure of 2.5 MPa were considered as the optimal process parameters.

Compared to pressed and milled PEEK, the 3D-printed PEEK material had lower HM parameters. However, the HM parameters of horizontally printed specimens are higher than those of vertically printed specimens [112]. Point-of-care (POC)-fabricated 3D-printed PEEK patient-specific implants (PSI) have been shown to have a high dimensional accuracy and reproducibility, exhibiting clinically acceptable morphological similarity in terms of fit and contour continuity [113]. The raw materials used in the process of AM can be mainly subdivided into liquid photopolymers, powders, and filaments [114]. Additionally, the ASTM Technical Committee classifies the current AM techniques into seven main categories, including the following: (i) powder bed fusion (PBF), (ii) material extrusion, (iii) vat photopolymerization, (iv) binder jetting, (v) material jetting, (vi) directed energy deposition, and (vii) sheet lamination [50]. The AM of PEEK materials is currently represented by technologies such as stereolithography (SLA), selective laser sintering (SLS), and FDM (Figure 2) [32]. Among them, SLA and SLS technologies, which are classified as vat photopolymerization and PBF technologies, use liquid resin and powder as raw materials, respectively. Meanwhile, FDM uses filament as feedstock and is classified as a material extrusion technology [50].

Among the seven AM technologies, PBF (Figure 2) has the greatest potential for large-scale production [115], but due to the high melting point of PEEK, the longest used 3D printing technique has primarily been SLS. The good mechanical properties of PAEK led to a rapid growth in the application of PAEK in PBF [116,117]. However, throughout the entire processing of PBF, the powder underwent non-isothermal crystallization [118], which can seriously affect the mechanical properties of the semicrystalline polymers such as PEEK. Yi et al. [21] synthesized a new type of PAEK copolymer, named PAEK2, by adding a polyetherdiphenyletherketone (PEDEK) comonomer, which is the first PAEK grade to achieve a high elongation (13%) while maintaining high crystallinity in the PBF process. The increase in molecular weight resulted in the slower crystallization of the new PAEK polymers and lower powder bed temperatures at 290 °C. The lower powder bed temperature is expected to reduce the level of thermal oxidative degradation and cross-linking, thus improving the recoverability of the powder and producing less shrinkage and warpage of the printed structure [119]. However, the powder particle size and morphology variations of PEEK make the sintering process complicated, which is a significant drawback of the technique.

FDM 3D printing technology has become a popular technology for manufacturing PEEK parts, as it has the advantage of carrying out customized and rapid manufacturing in small batches. However, the mechanical properties of most FDM 3D-printed general-purpose polymer parts struggle to meet the requirements of industrial applications due to weak interlayer adhesion [120]. The optimization of parameters such as FDM nozzle temperature and print orientation can result in printed PEEK parts with mechanical properties such as tensile strength, flexural strength, and impact strength that are approximately 80% of those of injection-molded parts [121]. Additionally, nozzle temperature is the largest parameter that affects the tensile properties of PEEK specimens fabricated with FDM 3D printers, as compared to the print layer thickness and printing speed [115]. In addition to the printing temperature, speed, orientation, and layer thickness, the properties of the filament used for FDM printing also affect the mechanical properties of the final printed specimen. As fast crystallization hinders interlayer diffusion, FFF rods printed with the slow-crystallizing novel PAEK copolymer achieve better particle coalescence with an improvement in the Z strength of around 40 MPa, which enhances the overall isotropy of the printed parts [122]. During the fusion filament manufacturing process, the high viscosity of PEEK can cause the buckling of filaments. The addition of inorganic fullerene tungsten sulfide (IF-WS) nanoparticles enhances the flow of PEEK and can reduce the melt viscosity of the polymer by 25% [100]. The reduction in viscosity promotes adhesion between printed layers without significantly increasing the melt temperature, which is beneficial in the FFF process. In addition to the nature of the filament itself, the postprocessing technology of the filament also has an impact on the mechanical properties of PEEK. It was found that postprocessing heat treatment (220 °C) of filaments fabricated using FFF facilitated the interfacial strength of the layers. The heat treatment significantly improved the modulus of elasticity (20%), tensile strength (45–65%), and fracture resistance (3–45%) of AM PEEK samples [123].

In oral and maxillofacial surgery, PEEK is successfully used for fracture fixation, maxillofacial bone repair, occlusal splints, and surgical guides for implant placement. Steffen et al. [124] found that PEEK plates did not seem to guarantee the displacement and mechanical integrity of mandibular reconstruction compared to miniature titanium plates. However, Avci et al. [125] concluded that the CF/PEEK plate/screw system reduces the stress on the fixation system and provides more stable fixation than the resorbable system. The 2 mm thick CF/PEEK plate seems to be a potential replacement for the 1 mm thick titanium plate when fixing dislocated mandibular fractures. In addition to its use in mandibular fracture fixation, PEEK is commonly processed into three-dimensional porous scaffolds for the treatment of massive bone defects due to its ability to control the volumetric geometry and internal junction of the tissue scaffold [126]. Uddin et al. [1] used melt casting and salt-based porogenic (200–500 micron size) leaching methods to fabricate highly porous, bionic PEEK bone scaffolds. The porosity of the scaffolds (75% and 85%) was adjusted by varying the salt concentration in the PEEK powder. HA, CF, and CNTs were used to improve the cell attachment and interaction with porous PEEK. A compression modulus enhancement of approximately 186% (252.91 MPa) and yield strength enhancement of 43% (4.51 MPa) was observed when only 0.5 wt.% CNTs were added to PEEK/HA. Using a low-temperature 3D printing process, Gao et al. [28] fabricated amorphous PAEK with carboxyl groups (PAEK-COOH) into hierarchically controllable porous scaffolds from the nanoscale to the microscale, with a mechanical strength comparable to that of trabecular bone. The nanoporous surface of the PAEK-COOH scaffold is beneficial in promoting cell adhesion, whereas the carboxyl groups can induce HA mineralization through electrostatic interactions. The scaffold provides better osseointegration than PEEK without additional active ingredients, providing a feasible method for the study of PEEK scaffolds in osteogenic applications.

### 3.4. Other Oral Applications of PEEK

Aesthetic orthodontic wires are another area of application for PEEK. Compared to other polymers, such as polyether sulfone (PES) and polyvinylidene fluoride (PVDF), PEEK orthodontic wires are able to deliver higher orthodontic forces, but they are at a similar cross-section of that of metallic wires such as CoCr, titanium-molybdenum (Ti-Mo), and nickel-titanium (Ni-Ti) [127]. In a self-ligating system, PEEK has comparable mechanical properties in load deflection to that of Ni-Ti wires [128]. Additionally, orthodontic wires covered with a PEEK tube can reduce friction, demonstrating a good combination of aesthetic and functional properties [129]. When used in a retainer following orthodontic treatment, PEEK can also provide a comparable performance to conventional retainers in terms of debonding and pull-out forces [130]. However, more research is needed on the mechanical properties and failure points of PEEK-bonded retainers [129].

Compared with metal implants, PEEK implants have better cartilage protection. Yuan et al. [131] used 3D printing and concentrated sulfuric acid to fabricate a porous sulfonated PEEK (SPK) scaffold, which matched the compressive modulus of the PEEK scaffolds (43 ± 5 MPa) to normal native cartilage (30 ± 8 MPa). SPK facilitates the growth and integration of new tissues and promotes the recovery of cartilage functions. In addition to the application of porous scaffolds, scaffolds with the precise incorporation of stem cells through 3D printing can cover periodontal bone defects and promote periodontal membrane (PDL) regeneration. Meanwhile, they can facilitate the absorption of masticatory pressure and provide micromotion to the teeth during mastication [132], which could be a potential direction for the future research and application of PEEK scaffold materials in dentistry (Table 4).

## 4. Adhesive Properties of PEEK in Dental Applications

PEEK, which is mostly jade white and ivory yellow, is unable to meet clinical aesthetic needs when used as a restoration in dentistry [15,16,45]. In order to achieve a color and translucency that is similar to natural teeth, resin veneers are often required for surface shading. Shear bond strength (SBS) values higher than 10 MPa between PEEK and resin-based composites have been reported to be clinically acceptable [133]. However, the hydrophobic surface and low surface energy of PEEK make it difficult to establish a strong and long-lasting bond. Therefore, PEEK material surface treatments and adhesive systems with resin are hot research topics with regard to the application of PEEK in the restorative field.

### 4.1. Surface Treatments

**Figure 3:**
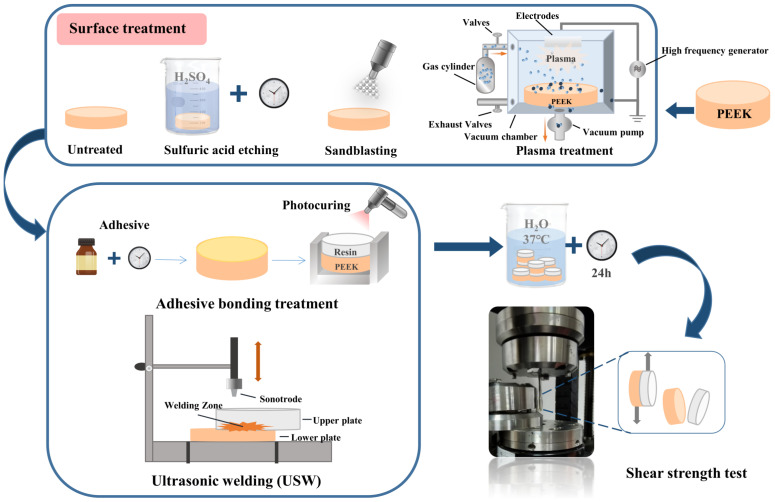
Schematic diagram of PEEK surface preparation and bonding performance testing. H_2_SO_4_—sulfuric acid.

The main methods of PEEK surface modification reported in current studies are sandblasting (SB; airborne particle abrasion); sulfuric acid (H_2_SO_4_) etching; plasma, UV/ozone, and radiation-induced (plasma gas, laser, electron, and ion beam) treatments; and chemical coating (Figure 3), which improve the wettability of PEEK and its bond strength to varying degrees (Table 5) [134].

Studies have reported that both the sandblast particle size (50 or 110 μm) and pressure can affect airborne particle abrasion treatments [145]. Adem et al. found no significant difference in SBS between PEEK treated with 50 μm Al_2_O_3_ particles at 2 MPa for 10 s (6.43 ± 1.05 MPa) and untreated PEEK (5.39 ± 1.36 MPa) [140]. However, a better bond strength was obtained by treating PEEK with 110 μm Al_2_O_3_ particles at 0.2 MPa for 15 s compared to untreated PEEK [15]. Compared with the simple sandblasting treatment, sandblasting with a silica coating with Al_2_O_3_ particles is an innovative surface modification method, but the SBS (8.07 ± 2.54 MPa) of silica-modified sandblasting (30 μm, 0.3 MPa, 15 s) was lower than that (10.81 ± 3.06 MPa) of simple sandblasting (110 μm, 0.28 MPa, 15 s) [145]. Sandblasting (50 μm, 0.25 MPa, 15 s) can also be treated in combination with an erbium-doped yttrium aluminum garnet (ER:YAG) laser treatment or oxygen plasma treatment, and the SBS treated in combination (22.0 ± 1.3 MPa; 21.2 ± 0.8 MPa) is much better than that from sandblasting alone (17.4 ± 2.4 MPa) [146]. However, the bond strength of 98% sulfuric acid etching alone for 1 min is higher than that of the acid etching group after sandblasting (50 μm, 2 MPa) [140]. This may be due to the fact that the high pressure of the surface sandblasting treatment causes the material to develop a highly porous and rough surface [138], which can adversely affect the penetration of the cement, thus weakening the adhesive interaction between PEEK and resin cement.

Acidic solutions such as sulfuric acid can increase the surface roughness of PEEK, ranging from 0.18 to 0.74 μm [144]. The modification of PEEK surfaces with 98% sulfuric acid has chemically and morphologically positive effects due to the formation of sulfonic acid groups within the highly porous and permeable outer layer [144] and the increased contact surface area, which leads to the enhanced mechanical interlocking of the adhesive and resin. Compared to the 50 μm alumina sandblasting treatment (1017.20 ± 53.70 N), the lithium-disilicate-veneered PEEK FDPs etched with 98% sulfuric acid had a higher load-bearing capacity (1040.25 ± 77.46 N) and were successful against physiological occlusal forces (216–847 N) [147]. Additionally, a synergistic effect between sulfonic acid functionalization and the acid adhesive was found when applying the universal (acidic) adhesive (Ambar Universal Adhesive, Brazil) on H_2_SO_4_-etched PEEK surfaces, which improved the bonding of PEEK to resin-based composite materials [139]. In addition to the type of adhesive, the difference in processing technology also has an impact on the bonding performance of the material. Chaijareenont et al. [144] reported that a good bond strength (27.36 MPa) was obtained after the etching of CAD/CAM-milled PEEK surfaces with 98% concentrated sulfuric acid for 60s, as it met the clinical requirements (ISO bond strength of 5 MPa). However, for 3D-printed PEEK, Zhang et al. concluded that the best bond strength (27.90 ± 3.48 MPa) could be obtained only after 30s of acid etching with concentrated sulfuric acid [143]. Moreover, the bonding properties of 3D-printed PEEK after acid etching with concentrated sulfuric acid were slightly lower than those of milled PEEK (SBS; >29MPa), but both were able to comply with the clinical requirements. The discrepancy in bonding properties may be attributed to the difference in characteristics, such as the internal crystalline structure and surface roughness of the material, caused by the two fabrication processes, the exact reasons for which need to be further explored.

Plasma treatments are also used to treat PEEK materials due to their safety and ease of handling. A low-pressure plasma treatment has the effect of etching and removing adherent particles to clean the surface and also affects the chemical structure of PEEK. The hydrogen-oxygen, 2/1-mixed plasma treatment that combined the action of hydrogen and oxygen plasma strongly improved the bond strength and the surface properties of the PEEK implant material, such as its crystallinity and surface microhardness [142]. Moreover, compared to another plasma treatment (argon and oxygen 1:1 gas mixture) or sandblasting (110 µm Al_2_O_3_ particles, 2.5 pressure), the highest overall SBS (19.8 ± 2.46 MPa) was observed in samples of both unfilled PEEK and pigment powder-filled PEEK compounds treated with low-pressure plasma after sandblasting [136]. However, the study by Bötel et al. [141] concluded that samples treated with a low-pressure oxygen plasma process after sandblasting, rather than an argon/oxygen plasma treatment, appeared to be most effective in improving the SBS (34.92 ± 6.55 MPa) between the veneer composite and the PEEK material.

The surface treatment of photodynamic therapy (PDT) relies on the formation of ROS and oxygen radicals to improve the surface energy of the PEEK surface [148]. Compared to surface treatments such as PDT (16.21 ± 0.14 MPa), H_2_SO_4_ (15.23 ± 0.63 MPa), and SB, Shabib et al. [138] observed that neodymium-doped yttrium orthovanadate (Nd:YVO_4_) laser-treated PEEK surfaces exhibited the highest SBS (16.33 ± 0.71 MPa) when bonded to composite resins. Another study [137] showed that the highest SBS was observed with 98% sulfuric acid (19.25 ± 0.68 MPa), whereas the bond strength of specimens treated with PDT was lower (11.69 ± 0.12 MPa). The different results of the two studies may stem from the difference in the type of adhesive and resin cement used for the experiments.

### 4.2. Adhesive Systems

After the surface treatment of the material, the influence of the type of adhesive system on the bond strength should also be considered. Oral restorative luting cements are mainly classified as zinc phosphate cement, zinc polycarboxylate cement, glass ionomer cement, and resin cements. Resin luting cements include universal resin cements and self-etching resin cements [12]. Self-etching resin cements (Multilink N, SBS:7.52 ± 1.20 MPa) exhibit a better bond strength than resin-modified glass ionomer cements (RMGIC) (RelyX Luting 2, SBS:3.85 ± 0.36 MPa) [149]. However, resin luting cements such as Panavia V5, RelyX^TM^ Ultimate Resin Cement, G-CEM Link Force, and Super-Bond C&B do not provide a sufficient SBS (<10 MPa) for PEEK [150]. Combining resin luting cement with sulfuric acid etching is a good strategy to improve the bonding behavior of PEEK. Additionally, regardless of the etching time, the bonding effect of universal resin cements (RelyX ARC and Variolink II) is better than that of self-adhesive resin cements (Clearfil SA Cement) [12].

The use of a bonding primer prior in the application of the luting cements can also affect the adhesive performance of PEEK. As probably due to the use of SE Bond, Zhou et al. found that the use of the dentin adhesive/composite resin cement (SE Bond/Clearfil AP-X™) resulted in a better bond strength than the use of a universal composite resin cement (Rely™ Unicem) [151]. SE Bond is a water-based primer that penetrates the porous surface of PEEK and is well suited for bonding the hydrophobic and chemically inert surfaces of PEEK [152]. Apart from this, the main bonding primers currently used to establish a bond between PEEK and resin matrix composites are Visio.link and Signum PEEK Bond [39]. Signum PEEK Bond contains a biofunctional monomer of methyl methacrylate (MMA) and phosphate ester groups, whereas the main components of Visio.link are MMA and pentaerythritol triacrylate [39]. Visio.link has a strong ability to modify the PEEK surface and enhance its micromechanical bond with the resin cement. Between the two adhesive systems, Visio.link demonstrated, overall, higher SBS values than Signum PEEK Bond and was preferred as an adhesive system [138]. Moreover, a sandblasting pretreatment improved the Visio.link bonding (SBS, 19.86 ± 2.52MPa), but did not affect Signum PEEK Bond [39].

To avoid the use of additional adhesives, it is also possible to weld PEEK denture brackets and veneer composites together based on the thermoplastic properties of PEEK [153,154]. The main welding methods for thermoplastic composites (TPCs) are thermal welding (infrared, hot tool, hot gas, extrusion, and laser), friction welding (vibration, rotation, splitting, and ultrasonic), and electromagnetic welding (induction, microwave, dielectric, and resistance) [153]. Abdulfattah et al. [135] used ultrasonic waves to convert mechanical vibrations of a low amplitude and high frequency into heat to weld different grades of PEEK samples (Figure 3). They found that different grades of PEEK samples had different optimal welding parameters. However, the increase in welding energy increased the surface deformation of all samples. Based on the promising applications of 3D printing materials, Khatri et al. [155] investigated the feasibility of the ultrasonic polymer welding of FFF manufactured PEEK and CF/PEEK with integrated energy directors. Ultimate lap-shear forces of 2.17 kN and 1.97 kN and tensile strengths of 3.24 MPa and 3.79 MPa were obtained for PEEK and CF/PEEK, respectively. This study minimizes the time and cost constraints of FFF as a manufacturing process while using ultrasonic welding as a glue-free method to join 3D-printed PEEK. However, the treatment of welding to achieve PEEK bonding is subject to more research.

In conclusion, the choice of PEEK surface treatment can be combined with different adhesive systems, but this should be subjected to further clinical research and specifications. PEEK composites such as CF/PEEK and HA/PEEK exhibit excellent biocompatibility due to the inherent bioactivity of the reinforcing material [44]. However, relatively few studies have focused on the application of these novel materials in improving adhesion. Moreover, most bioactive materials are encapsulated in PEEK matrices and the surface bioactivity of these composites is still low. Researchers often modify the surface of PEEK composites to improve their surface bioactivity through roughening treatments [17] and the introduction of chemical groups [18] and coatings [156]. Among them, biomolecular modifications such as the extracellular matrix (ECM), growth factors (GFs), bioactive peptides, plant extracts, and antibiotics [52] can enhance the antimicrobial properties and osteogenic activity of PEEK and its composites. Advances in the research on the antimicrobial properties and osteogenic activity of PEEK and its composites are less discussed in this paper and are the limitations of this review.

## 5. Conclusions and Perspectives

PEEK has many advantages in dentistry, and, for patients with a weak tolerance to stress and sensitivity to metallic materials, PEEK and its composites can be an excellent implant and restorative material. However, it is unclear which manufacturing method is best suited for dental applications of PEEK. For example, 3D printing can produce structurally complex restorations with superior mechanical properties [20], but the accuracy of this manufacturing method must be further investigated. Additionally, 3D printing processing techniques and postprocessing processes can affect the crystallinity of PEEK materials. A small degree of crystallinity may lead to deficiencies in the mechanical properties of the material [122], whereas a large degree may lead to deformation [99,100]. The effect of crystallinity on the mechanical properties and deformation of the material needs to be further studied to achieve a balance of properties. In addition to excellent mechanical properties, promising clinical implants should have good biocompatibility, significant osteoconductive and osteoinductive activity, and excellent anti-inflammatory and anti-infective properties [18]. Researchers need to further explore the development of a new generation of PEEK materials with good mechanical properties, osseointegration capabilities, and antimicrobial properties.

In terms of bonding properties, most of the available studies on the bonding behavior of PEEK have only been performed in vitro, and the clinical testing of molded specimens is lacking. Given the complexity of the oral environment, future studies should focus on the effects of the oral environment on the bond strength and bond microleakage to assess the clinical viability and long-term performance of PEEK. The effects of different fabrication techniques and technical parameters, such as printing temperature, speed, and layer thickness, on the adhesive properties of PEEK also warrant further clinical studies. Overall, the clinical performance of PEEK materials is satisfactory and promising. Future large-scale and long-term controlled clinical studies should be conducted to obtain more useful outcomes of PEEK in dental application.

## Figures and Tables

**Table 1:** Performance requirements of medical materials and related test methods.

Properties	Requirements	Brief Introduction	Test Methods	Ref.
Mechanical properties	Elastic modulus (i.e., Young’s modulus, stiffness; GPa)	The ability to resist elastic deformation. It is defined as the ratio of stress to strain and is determined from the initial slope of the stress–strain curve.	- Tensile test - Bending test - Compressive test	[7]
	Tensile strength (MPa)	The ultimate strength of the material during tension.	Tensile test	[20]
	Elongation (%)	The ability to stretch plastically (i.e., ductility).	Tensile test	[21]
	Elongation at break (%)	The ratio between the length of a specimen that changes after fracture and the initial length.	Tensile test	[21]
	Bending strength (MPa)	The ultimate strength of the material during bending.	Bending test	[20]
	Compressive strength (MPa)	The ultimate strength of the material during compression.	Compressive test	[22]
	Shear strength (interlaminar and interfacial shear strength (ILSS, IFSS); MPa)	The resistance of the composite to delamination under shear forces parallel to the layers of the laminate, and thus, to the adhesive/adherent interface.	- Shear bonding strength test - Pull-off tensile test	[23,24]
	Yield strength (MPa)	The ability to withstand stress without plastic deformation (i.e., permanent deformation).	- Tensile test - Compressive test	[21]
	Toughness (KJ/m^2^)	The energy of elastic and plastic deformation required to break a material. It increases with strength and ductility.	Impact test	[23]
	Fatigue strength (S_n_; MPa)	The maximum alternating stress that the material can withstand for a long time.	Load-cycle fatigue test	[25]
	Creep	Under the condition of a constant load below the yield strength, the strain increases with time.	Martens force/depth indentation test	[26]
	Hardness (indentation hardness, scratch hardness, rebound hardness; MPa)	The ability of a material surface to resist plastic deformation caused by indentation and localized cracking. Rebound hardness means the magnitude of the elastic deformation work of the material.	- Vickers hardness (HV) test - Martens hardness (HM) test - Surface scratch test - Back-jump test	[19]
Biological properties	Biocompatibility	The ability of a material to generate an appropriate host response in a specific application.	Cell proliferation, cytotoxicity, and adhesion test	[27]
	Osseointegration	A phenomenon where an implant becomes fused with bone.	Microcomputed tomography (μ-CT)	[28]
Chemical properties	Corrosion resistance	The ability to resist damage caused under the action of the surrounding medium.	Potentiodynamic polarization (PDP) and static immersion assay	[29]
	Aging resistance	The ability of polymers to resist deterioration.	Cycles of thermal aging	[26]
Physical characteristics	Crystallinity	A specific type of ordered structure in a solid material. Those with little crystallinity are known as amorphous polymers.	Differential scanning calorimetry (DSC) and X-ray diffraction (XRD)	[21]
	Microscopic features	Surface, cross-sectional, and fracture surface characteristics, such as roughness.	Scanning electron microscopy (SEM)	[26,30]
	Porosity	The ratio of the void volume to total volume.	Water displacement method	[30]
	Surface tension (N/m)	The ability to cause the surface of a liquid to shrink.	Water contact angle detection	[30]

**Table 2:** Advantages and disadvantages of materials used in dentistry. PEEK: polyetheretherketone; PMMA: polymethylmethacrylate; FDPs: fixed dental prostheses.

Material Characteristics	Materials	Advantages	Disadvantages	Dental Application	Ref.
Metal-based materials	Titanium and its alloys (Ti-6Al-4V)	- Improved strength - Biocompatible	- High Young’s moduli - Poor wear resistance - Potentially toxic	- Dental implants and abutments - Inapplicable for orthopedic use (Ti-6Al-4V)	[33]
	Cobalt-based alloys (cobalt-chromium-molybdenum/CoCr-Mo)	- Low rigidity - High yield and tensile strength - Superior wear resistance	- Unaesthetic appearance - Potentially toxic	- Ceramic abutments - Crowns - Clasps	[34]
Ceramics	Alumina (Al_2_O_3_) (α-aluminum oxide)	- Biocompatible - Wear resistant	- Less compact - Lower flexural strength	- Dental implants - Endodontic posts - Orthodontic brackets	[35]
	Zirconia (ZrO_2_)	- Highly biocompatible - Good osseointegration - Good aesthetics	High Young’s moduli	- Crowns - Implant abutments	[34]
	Lithium disilicate	Superior aesthetics and translucency	High Young’s moduli	Crowns	[36]
	Titanium dioxide (TiO_2_) nanoparticles	Semipermanent antibacterial agent	Resulting in cytotoxicity in a dose-dependent manner	Oral antibacterial disinfectants, whitening agents, and adhesives	[37,38]
Polymers	PEEK	- Good mechanical properties - Good biocompatibility	- Poor surface properties - Poor aesthetic performance	- Dental abutments - Temporary crowns	[7]
	PMMA	- Non-biodegradable and stable aesthetic - Good flexibility	Shrink during polymerization	- Denture base - Crowns	[39]
Composites	Carbon-fiber-reinforced PEEK (CF/PEEK)	Higher mechanical strength and wear resistance than PEEK	Relatively weak interlaminar strength	-Fracture fixation -Posts and cores	[40]
	Glass-fiber-reinforced PEEK (GF/PEEK)	Higher rigidity, hardness, and deformation resistance	Poor uniformity	Posts and cores	[41]
	PEEK /nano-silica (PEEK/nano-SiO_2_)	Higher elastic modulus	Decreased toughness	Crowns	[42]
	Hydroxyapatite (HA)/PEEK (HA/PEEK)	Increased compressive strength and modulus with the HA content	Decreased tensile/bending strength	Bone grafting and tissue engineering scaffolds	[43,44]
	TiO_2_-reinforced PEEK	- Higher fracture and aging resistance - Improved Martens hardness (HM)	Affected by radiation	Implant-supported, 4-unit cantilever FDP	[45]
	Polymer-infiltrated ceramic network (PICN)	Comparable fracture toughness and better damage tolerance than glass ceramics	Significantly lower wear resistance than that of tooth enamel	Dental restorations for bruxism patients	[34]

**Table 3:** The mechanical properties of oral tissue, common medical materials, and PEEK. CoCr: cobalt-chromium; FDM: fused deposition modeling.

Materials	Density (g/cm^3^)	Martens Hardness (HM, N/mm^2^)	Tensile Strength (MPa)	Elastic Modulus (GPa)	Bending Strength (MPa)	Ref.
Cortical bone	1.92		104–121	6–30	225	[46]
Dentin	3.3	468.2 ± 30.77	104	12–18.6		[47]
Dental enamel		2263.6 ± 405.16	47.5	40–83		[47]
Titanium	4.5	300–400	954–976	102–110		[47]
CoCr	6.5	1200	680	205	800–1400	[48]
PMMA	1.18	180	48–76	3.6	95–105	[47]
PEEK	1.3	189.55 ± 16.89	87.53–100	3–4	99.25–170	[1,22]
Annealed FDM-printed PEEK			97.34	2.6–3.45	104.65	[22,49]
PEEK/CF (carbon-fiber-reinforced PEEK)	1.3	330.6 ± 21.2	6.9–109	3.5–58.5	264.6	[50]
PEEK/GF (30% glass-fiber-reinforced PEEK)	2.61	295.3 ± 12.5	94.0 ± 2.0	12.38	167	[26]
Bio-PEAK (PEEK filled with 5% TiO_2_)	1.4			4.2–4.8	190–210	
Dentokeep PEEK (PEEK filled with 20% TiO_2_)	1.5	191.45 ± 15.49	83.1	4.24		[47]
breCAM.BioHPP (PEEK filled with 20–30% TiO_2_)	1.3	197.35 ± 19.9		4.2	160	[45]
PEEK 450G (PEEK filled with 30% TiO_2_)	1.3	142 ± 34.7	95.21 ± 1.86	5.05	163	[50]
PEEK composite containing 20–30% HA	1.28		49–59	5–7		[51]

**Table 4:** Recent mechanical property advances of PEEK in oral applications. CFR-PEEK: carbon-fiber-reinforced PEEK.

Application	Subcategory	Materials Tested	Outcomes	Ref.
Implant therapy	Implant	- Titanium implant - Ti-PEEK composite implant	Ti-PEEK composite implant was superior in reducing bone resorption (stress shielding).	[54]
		- 2–4 mm HA particles in PEEK - Nanosized HA particles in PEEK	Implants made from PEEK nanocomposite sites have better mechanical properties.	[57]
	Abutments	- PEEK - Grade 5 titanium	PEEK abutments are suitable for long-term provisional restorations in the anterior part with no functional impairment.	[31]
		- Custom PEEK healing abutment - Standard healing caps	The custom PEEK healing abutment created a natural gingival structure and required fewer steps to create an emergency contour.	[7,60]
		- Zirconia/lithium silicate/resin-based ceramic/PEEK crowns - Zirconia/PEEK abutment	High-strength rigid zirconia and lithium disilicate ceramics benefit more from a favorable stress distribution when applied on PEEK abutments.	[63]
		- Straight/15° angle/25° angle abutment - Porcelain metal (PFM)/PEEK restoration crowns	Molar patients can be given straight abutments combined with PEEK crowns to reduce intraosseous stress concentration.	[65]
Prosthodontic therapy	Crown	- Milled crowns (PEEK, PMMA, and silicate ceramic) - Crown–abutment material combinations	PMMA crowns showed the highest material loss and PEEK had the lowest material loss.	[24]
	Implant-supported, 4-unit fixed restorations	- 20%/30% TiO_2_-filled PEEK - Veneered resin composite/digital veneering/prefabricated veneering	The highest fracture resistance of the restorations was achieved when 30% TiO_2_-filled PEEK material was used and prefabricated veneers were applied.	[24,45]
	Partial dentures	- PMMA - PEEK	PEEK provides a higher Young’s modulus but lower flexural deformation than PMMA, which may reduce the load applied to the underlying tissue.	[81]
	Clasp	- Shape-optimized PEEK clasp - Standard shape CoCr clasp	-The retention forces provided by the PEEK clasp were adequate for clinical use. There was no significant difference in long-term deformation between the two materials.	[85]
Oral and maxillofacial surgery	Mandibular fracture fixation	- CFR-PEEK plate/screw system - Resorbable system	CFR-PEEK plate/screw system reduces the stress on the fixation system and provides more stable fixation.	[125]
	Bone scaffolds	- PEEK - PAEK with carboxyl groups (PAEK-COOH)	PAEK-COOH controllable porous scaffolds had better mechanical strength and are beneficial for promoting cell adhesion.	[28]
Other oral applications	Orthodontic wires	- PEEK - Polyether sulfone (PES) - Polyvinylidene fluoride (PVDF)	PEEK orthodontic wires are able to deliver higher orthodontic forces, but at a similar cross-section of that of metallic wires.	[127]
	Cartilage recovery	- PEEK - Sulfonated PEEK (SPK)	SPK favors the secretion of anti-inflammatory cytokines and promotes the recovery of cartilage functions.	[131]

**Table 5:** The adhesive properties of PEEK bonded with composite resin. H_2_SO_4_: sulfuric acid.

Material	Surface Treatment	Adhesive	Shear Bonding Strength (SBS; MPa)	Surface Roughness (Ra; μm)	Ref.
PEEK	Untreated	Ultrasonic welding (USW)	16.37 ± 1.69		[135]
	Untreated	Visio.link	3.81 ± 2.71	0.69 ± 0.07	[136]
	98% H_2_SO_4_ etching for 60 s	Silane coupling agent	19.25 ± 0.68	2.658 ± 0.658	[137]
	98% H_2_SO_4_ etching for 60 s	Visio.link	15.23 ± 0.6	0.61 ± 0.14	[138]
	98% H_2_SO_4_ etching for 60 s	Ambar Universal Adhesive	17.84 ± 2.8	1.05 ± 0.59	[139]
	98% H_2_SO_4_ etching for 60 s after Al_2_O_3_ sandblasting (50 μm, 2 MPa, 10 s)	Visio.link	11.72 ± 1.69	-	[121]
	Al_2_O_3_ sandblasting (50 μm, 2 MPa, 10 s)	Visio.link	6.43 ± 1.05	-	[140]
	Al_2_O_3_ sandblasting (110 μm, 0.1 MPa, 10 s)	Silane coupling agent	14.55 ± 1.25	1.552 ± 0.002	[137]
	Al_2_O_3_ sandblasting (110 μm, 0.1 MPa)	Visio.link	10.71 ± 0.52	0.92 ± 0.12	[138]
	Al_2_O_3_ sandblasting (110 μm, 2.5 MPa)	Visio.link	18.29 ± 1.84	1.64 ± 0.48	[136]
	Oxygen plasma treatment for 3 min	Visio.link	21.65 ± 5.31	0.69 ± 0.22	[141]
	Hydrogen–oxygen, 2/1-mixed plasma treatment	-	-	0.43 ± 0.06	[142]
	Argon and oxygen 1:1 process for plasma treatment	Visio.link	3.76 ± 2.42	0.06 ± 0.07	[136]
	Argon and oxygen 1:1 process for plasma treatment after sandblasting (110 μm, 2.5 MPa)	Visio.link	19.8 ± 2.46	1.32 ± 0.39	[136]
	Photodynamic therapy (PDT)	Silane coupling agent	11.69 ± 0.12	1.254 ± 0.011	[137]
	Photodynamic therapy (PDT)	Visio.link	16.21 ± 0.14	14.25 ± 1.21	[138]
	Neodymium-doped yttrium orthovanadate (Nd: YVO_4_) laser treatment	Visio.link	16.33 ± 0.71	15.25 ± 1.58	[138]
3D-printed PEEK	98% H_2_SO_4_ etching for 30 s	Visio.link	27.90 ± 3.48	-	[143]
CAD/CAM-milled PEEK	98% H_2_SO_4_ etching for 60 s	Visio.link	27.36	0.74 ± 0.25	[144]
	98% H_2_SO_4_ etching for 5–120 s	Visio.link	>29	-	[143]
Vestakeep DC4420 (PEEK filled with 20% TiO_2_)	Argon and oxygen 1:1 process for plasma treatment after sandblasting	Visio.link	15.86 ± 4.39	1.19 ± 0.4	[136]
	Oxygen plasma treatment for 3 min	Visio.link	30.95 ± 6.35	2.0 ± 0.97	[141]
DC 4450 (filled with 20% TiO_2_ powder and 1% pigment)	Argon and oxygen 1:1 process for plasma treatment after sandblasting	Visio.link	9.06 ± 3.1	1.83 ± 0.17	[136]
	Oxygen plasma treatment for 3 min	Visio.link	34.92 ± 6.55	0.93 ± 0.3	[141]
breCAM.BioHPP	Silica-modified sandblasting (30 μm, 0.3 MPa, 15 s)	Visio.link	8.07 ± 2.54	0.42 ± 0.03	[145]
	Al_2_O_3_ sandblasting (110 μm, 0.2 MPa, 15 s)	Visio.link	10.81 ± 3.06	2.26 ± 0.33	[145]
	Al_2_O_3_ sandblasting (50 μm, 0.25 MPa, 15 s)	Bond.lign	17.4 ± 2.4	2.1 ± 0.2	[146]
	Oxygen plasma treatment after sandblasting (50 μm, 0.25 MPa, 15 s)	Bond.lign	21.2 ± 0.8	2.7 ± 0.1	[146]
	Erbium-doped yttrium aluminum garnet (ER: YAG) laser treatment after sandblasting (50 μm, 0.25 MPa, 15 s)	Bond.lign	22.0 ± 1.3	2.9 ± 0.1	[146]

## Data Availability

Not applicable.
